# Neurodevelopmental Disorders and the Inverse Metabolic Profile: A Nationwide Propensity-Matched Analysis

**DOI:** 10.7759/cureus.108191

**Published:** 2026-05-03

**Authors:** Edgar M Luna Landa, Melina Arroyo Cisneros, Gloria Erazo, Juan Pedroza, Guy Nguefang

**Affiliations:** 1 Internal Medicine, Texas Tech University Health Sciences Center, Odessa, USA; 2 Biomedical Engineering, Universidad Autonoma de Queretaro, Queretaro, MEX; 3 Medicine, Universidad Autonoma de Guadalajara, Guadalajara, MEX

**Keywords:** autism spectrum disorder, intellectual disability, malnutrition, national inpatient sample, neurodevelopmental disorders, non-alcoholic fatty liver disease, obesity

## Abstract

Background

Adults with neurodevelopmental disorders (NDD) may exhibit a distinct metabolic and nutritional risk profile compared to the general population. Although pediatric studies have reported higher obesity rates in certain neurodevelopmental disorder subtypes, particularly autism spectrum disorder, findings are heterogeneous, and national-level data in adults remain limited. This study assessed the association between NDD and three key metabolic outcomes in hospitalized adults: obesity, non-alcoholic fatty liver disease (NAFLD), and protein-calorie malnutrition.

Methods

This retrospective study utilized the National Inpatient Sample (2016-2020) and included adults aged 18 years and older. NDD was identified using diagnostic codes for autism spectrum disorder, intellectual disability, and related developmental disorders. The primary outcomes were obesity, NAFLD, and protein-calorie malnutrition. All hospitalizations involving NDD were included. To ensure an adequate pool of potential controls for matching, a 20:1 random sample of non-NDD hospitalizations was initially selected, followed by 1:4 nearest-neighbor propensity score matching based on age, sex, race, primary payer, and income quartile. Survey-weighted multivariable logistic regression models were used to estimate adjusted odds ratios (aORs).

Results

The final matched cohort comprised 856,315 weighted hospitalizations, including 171,263 adults with NDD and 685,052 matched controls. Propensity score matching achieved excellent covariate balance, with all standardized mean differences <0.10, indicating clinically similar baseline characteristics between groups. Adults with NDD demonstrated an inverse metabolic profile, with lower odds of obesity (aOR 0.76) and NAFLD (aOR 0.56), but higher odds of protein-calorie malnutrition (aOR 1.25).

Conclusions

Hospitalized adults with neurodevelopmental disorders exhibit an inverse metabolic profile, characterized by lower odds of obesity and NAFLD but higher odds of malnutrition. These findings contrast with the pediatric literature and indicate that metabolic risk patterns in NDD may shift with age. Recognition of this distinct profile may inform targeted metabolic screening and nutritional interventions for this vulnerable population.

## Introduction

Neurodevelopmental disorders (NDD) are a heterogeneous group of lifelong conditions that begin during the developmental period and are characterized by impairments in cognition, communication, behavior, and adaptive functioning. NDDs are primarily defined by functional and behavioral constructs, with multiple definitions that vary substantially across clinical, administrative, and research settings [1,2]. Intellectual disability and autism spectrum disorder are among the most common conditions included within this category.

Obesity and nonalcoholic fatty liver disease (NAFLD) are well-characterized metabolic conditions in the general United States population [3]. However, the metabolic profile of adults with NDD remains insufficiently described. Adults with NDD often face unique nutritional and feeding challenges, such as dysphagia, food aversions, and reliance on caregivers for meals [4-6]. These factors may affect their nutritional status in ways that differ from the general population.

Despite this knowledge gap, limited data exist regarding obesity, fatty liver disease, and protein-calorie malnutrition among hospitalized adults with NDD. Previous research has primarily focused on obesity prevalence in pediatric populations with NDD [7]. To address this limitation, we conducted a national analysis to compare the relative burden and adjusted odds of obesity, fatty liver disease, and protein-calorie malnutrition among hospitalized adults with NDD and a matched non-NDD population.

## Materials and methods

Study design

We conducted a retrospective study using the National Inpatient Sample (NIS), developed and maintained by the Healthcare Cost and Utilization Project (HCUP) of the Agency for Healthcare Research and Quality (AHRQ) from 2016 to 2020 [8]. The NIS is a large nationwide database including approximately 20% of US hospitalizations across all 50 states, including patient and demographic characteristics, hospital type, insurance status, hospital length of stay, and healthcare costs. NIS patient hospitalizations are weighted to provide nationally representative estimates using survey weights [9]. As the NIS database contains de-identified data, this study was exempt from institutional review board approval or exemption determination.

Study population

The study population consisted of adult patients (aged 18 years or older) hospitalized with a diagnosis of intellectual disability, autism spectrum disorder, or other specified neurodevelopmental disorders, identified using ICD-10-CM codes [10] listed in Supplementary Table 3. 

Hospitalizations with missing data for key covariates, including age, sex, race/ethnicity, insurance status, income quartile, length of stay, or mortality, were excluded. We excluded patients with Down syndrome and cerebral palsy, given the well-established and distinct metabolic profiles described in these populations, which could confound the analysis.

Exposure group and outcomes

The primary exposure was hospitalization with NDD. The primary outcomes were obesity/overweight, fatty liver disease, and protein-calorie malnutrition (including kwashiorkor and marasmus). These outcomes were identified using ICD-10-CM diagnostic codes listed in Supplementary Table 3.

Control group (non-NDD)

The control group comprised adult hospitalizations without any of the specified NDD diagnosis codes.

Statistical analysis

Data extraction and initial processing were performed using Structured Query Language (SQL). To ensure a computationally feasible yet adequately sized control pool for matching, all eligible NDD hospitalizations were retained, and a 20:1 random sample of non-NDD hospitalizations was selected for potential matching.

Propensity score matching (PSM) was performed using a nearest-neighbor algorithm without replacement at a 1:4 ratio, matching each NDD hospitalization to four unique non-NDD controls. Propensity scores were estimated using age, sex, race/ethnicity, primary payer, and median household income quartile. This matching ratio was chosen to maximize statistical power while maintaining balance across covariates.

Post-matching balance between groups was assessed using standardized mean differences and p-values for baseline characteristics. After successful matching, multivariable logistic regression models estimated adjusted odds ratios (aORs) with 95% confidence intervals (CIs) for the association between NDD status and each metabolic outcome. All statistical analyses were conducted using R software (R Foundation, Vienna, Austria). A two-sided p-value less than 0.05 was considered statistically significant.

## Results

Study demographics

A total of 856,315 weighted adult hospitalizations were included in the final propensity-score matched analysis, comprising 171,263 patients with Neurodevelopmental Disorders (NDD) and 685,052 non-NDD controls.

Table 1 summarizes the baseline demographic and socioeconomic characteristics of the matched cohort. The mean age was similar between groups (46 ± 18 years for NDD and 47 ± 18 years for controls). The NDD group had a higher proportion of male (61% vs. 57%) and White patients (70% vs. 67%); however, standardized mean differences (0.08 and 0.07, respectively) indicated adequate covariate balance. Socioeconomic indicators were comparable, with the largest proportion of patients in both cohorts residing in the lowest income quartile (32% of NDD and 34% of controls) and using Medicare as the primary payer (58% of NDD and 54% of controls). Although these small absolute differences were statistically significant due to the large sample size (p < 0.001), the overall distributions were clinically similar.

**Table 1:** Baseline demographics * Age presented as mean ± standard deviation; categorical variables presented as n (%). Values <0.10 indicate adequate covariate balance. NDD - neurodevelopmental disorders; SMD - standardized mean difference

Characteristic	Non-NDD (n=685,052)	NDD (n=171,263)	SMD
Age*	47 ± 18	46 ± 18	0.06
Gender			0.08
Male	391,574 (57%)	104,312 (61%)	
Female	293,478 (43%)	66,951 (39%)	
Race			0.07
White	458,238 (67%)	119,739 (70%)	
Black	134,013 (20%)	29,734 (17%)	
Hispanic	60,632 (8.9%)	13,869 (8.1%)	
Asian or Pacific Islander	9513 (1.4%)	2539 (1.5%)	
Native American	3923 (0.6%)	848 (0.5%)	
Other	18,733 (2.7%)	4534 (2.6%)	
Primary payer			0.09
Medicare	368,459 (54%)	99,309 (58%)	
Medicaid	218,059 (32%)	48,354 (28%)	
Private insurance	77,874 (11%)	18,820 (11%)	
Self-pay	9084 (1.3%)	2288 (1.3%)	
No charge	607 (<0.1%)	161 (<0.1%)	
Other	10,969 (1.6%)	2331 (1.4%)	
Median household income quartile			0.06
Lowest (Q1)	235,483 (34%)	54,729 (32%)	
Low-middle (Q2)	188,134 (27%)	47,094 (27%)	
Upper-middle (Q3)	149,961 (22%)	38,752 (23%)	
Highest (Q4)	111,474 (16%)	30,688 (18%)	

Primary metabolic outcomes

In the propensity-matched cohort, logistic regression analysis identified a distinct metabolic profile in the NDD population, with significantly lower odds of obesity (aOR 0.76; 95% CI 0.75-0.78) and NAFLD (aOR 0.56; 95% CI 0.54-0.59), but higher odds of protein-calorie malnutrition (aOR 1.25; 95% CI 1.22-1.27). 

As shown in Table 2, adults with NDD had significantly lower adjusted odds of obesity and non-alcoholic fatty liver disease, but higher odds of protein-calorie malnutrition compared with matched controls.

**Table 2:** Analysis of metabolic outcomes Models adjusted for age, sex, race, primary payer, and income quartile. Reference group: Non-NDD matched controls. NDD - neurodevelopmental disorders

Outcome	Adjusted OR (NDD vs. control)	95% Confidence Interval	p-value
Obesity	0.76	0.75 – 0.78	<0.001
Non-alcoholic fatty liver disease	0.56	0.54 – 0.59	<0.001
Protein-calorie malnutrition	1.25	1.22 – 1.27	<0.001

## Discussion

To our knowledge, this study represents the first nationwide, propensity-matched analysis to characterize the metabolic and nutritional phenotype of hospitalized adults with neurodevelopmental disorders (NDD). This nationally representative cohort revealed a distinct inverse metabolic profile in this population. In contrast to prevailing trends in the general United States population, where metabolic syndrome is the primary concern [11], adults with NDD demonstrated significantly lower odds of obesity and non-alcoholic fatty liver disease (NAFLD). However, this apparent metabolic protection was accompanied by a 25% higher risk of protein-calorie malnutrition. These findings indicate that metabolic risks associated with NDD may change across the lifespan and highlight the importance of prioritizing nutritional screening over metabolic syndrome surveillance in the adult inpatient setting.

The observation that adults with NDD have lower odds of obesity (aOR 0.76) and fatty liver disease (aOR 0.56) contrasts with findings from the pediatric literature, where elevated obesity rates are frequently reported compared to neurotypical children [7]. This difference may indicate a metabolic shift as individuals with NDD transition into adulthood. Several factors could contribute to this phenomenon. Adults with severe NDD often reside in group homes or institutional settings, which may protect them from the obesogenic environment of the general population [12]. Our finding of lower obesity contrasts with a previous study in community-dwelling older adults with NDD, which reported higher obesity rates compared to the general population [13]. This discrepancy supports the hypothesis that hospitalized adults with NDD may represent a select subset with greater medical complexity, distinct from the broader community population [14]. Therefore, our findings should not be generalized to non-hospitalized adults with NDD. The reduced odds of NAFLD are likely driven by the lower prevalence of obesity in our study population, given the established pathophysiological link between visceral adiposity and hepatic steatosis [15,16].

Despite the lower burden of metabolic disease, this should not be interpreted as evidence of optimal nutritional health. Our analysis demonstrated that adults with NDD are significantly more susceptible to deficiency, with a 1.25-fold higher odds of protein-calorie malnutrition. This result aligns with the established pathophysiology of NDD, which frequently includes oropharyngeal dysphagia, sensory-based feeding aversions, and gastrointestinal dysmotility [5,6]. In the acute inpatient setting, these individuals are at increased risk for iatrogenic malnutrition due to prolonged NPO status, dysphagia, and communication barriers [17-19]. The observed inverse metabolic profile highlights the risk that standard general-population screening protocols, which focus on weight loss and cholesterol, may overlook occult malnutrition, the primary driver of morbidity found in this population. While population-based studies identify cardiovascular disease as the leading cause of death in the general adult population [20], the lower odds of obesity and NAFLD observed in our NDD cohort raise the possibility that their mortality profile may differ. Whether malnutrition-related complications (e.g., infections, frailty) outweigh cardiovascular risks in this population remains unknown and warrants longitudinal investigation.

Strengths and limitations

The primary strength of this study is the use of the National Inpatient Sample, which enables a highly powered analysis generalizable to the US hospital system. Additionally, the 1:4 propensity score matching technique identified an independent association between NDD and metabolic outcomes while controlling for key socioeconomic determinants of health, such as income and insurance status. Our study also has limitations inherent to retrospective administrative database research. First, reliance on ICD-10 codes may result in under-reporting of milder cases and potential misclassification bias. Malnutrition codes, in particular, may preferentially capture severe cases requiring clinical intervention, potentially overestimating the true association. Second, we deliberately restricted our NDD definition to intellectual and developmental disorders (F-codes), excluding primary motor disorders such as cerebral palsy. This decision strengthens internal validity by removing the potential confounder of immobilization-related metabolic changes. While this strengthens our conclusions regarding the cognitive/behavioral NDD phenotype by removing the confounder of immobilization-related atrophy, our findings may not apply to patients with primary motor disabilities. Finally, because this is a cross-sectional analysis, we cannot determine causality between NDD status and nutritional outcomes.

## Conclusions

Hospitalized adults with neurodevelopmental disorders demonstrate a distinct inverse metabolic profile, characterized by significantly lower odds of obesity and non-alcoholic fatty liver disease, but a higher risk of protein-calorie malnutrition compared to matched controls. This pattern contrasts with pediatric findings of increased obesity risk, raising the possibility that metabolic vulnerability in NDD may differ between children and hospitalized adults. Longitudinal studies are needed to confirm whether metabolic profiles change with age. The increased prevalence of malnutrition constitutes an underrecognized clinical risk that may contribute to adverse health outcomes, functional decline, and greater healthcare utilization, although these downstream consequences were not directly assessed in this study.

These findings underscore the necessity for routine nutritional assessment and individualized metabolic screening strategies in hospitalized adults with neurodevelopmental disorders. Routine nutritional screening should be incorporated into standard inpatient care for adults with NDD. Further longitudinal and mechanistic research is needed to elucidate the biological, behavioral, socioeconomic, and healthcare access factors that may contribute to this unique metabolic phenotype. Enhanced understanding of these pathways could inform targeted interventions to optimize long-term metabolic and nutritional outcomes in this vulnerable population.

## Appendices

**Table 3:** Supplementary table

Variable category	Diagnosis description	ICD-10-CM codes included
EXPOSURE: Neurodevelopmental disorders (NDD)	Intellectual disabilities	F70–F79 (All sub-codes) • F70: Mild intellectual disabilities • F71: Moderate intellectual disabilities • F72: Severe intellectual disabilities • F73: Profound intellectual disabilities • F78: Other intellectual disabilities • F79: Unspecified intellectual disabilities
	Autism spectrum disorders	F84 (All sub-codes) • F84.0: Autistic disorder • F84.2: Rett's syndrome • F84.5: Asperger's syndrome • F84.8: Other pervasive developmental disorders • F84.9: Pervasive developmental disorder, unspecified
	Unspecified psychological development	F88: Other disorders of psychological development F89: Unspecified disorder of psychological development
OUTCOMES	Obesity & overweight	E66 (All sub-codes) • E66.0: Obesity due to excess calories • E66.01: Morbid (severe) obesity • E66.1: Drug-induced obesity • E66.3: Overweight • E66.8: Other obesity • E66.9: Obesity, unspecified
	Non-alcoholic fatty liver disease (NAFLD)	K76.0: Fatty (change of) liver, not elsewhere classified K75.81: Nonalcoholic Steatohepatitis (NASH)
	Protein-calorie malnutrition	E40–E46 • E40: Kwashiorkor • E41: Nutritional marasmus • E43: Unspecified severe protein-calorie malnutrition • E44: Protein-calorie malnutrition of moderate and mild degree • E45: Retarded development following protein-calorie malnutrition
